# A Review of Novel Bacterial Complex Lipids: Implications for the Pathogenesis of Apical Periodontitis

**Published:** 2010-11-15

**Authors:** Qiang Zhu

**Affiliations:** 1. Department of Endodontics, School of Dental Medicine, University of Connecticut Health Center, Farmington, CT, USA.

**Keywords:** Periapical Periodontitis, Bacterial Lipids, Sphingolipid, Toll-Like Receptors, Bone Resorption

## Abstract

The importance of the role played by bacteria in the pathogenesis of pulpal and apical disease has been established. One of the characteristics of apical periodontitis is apical bone resorption, which is due to apical immune response to bacterial infection. Recently, novel bacterial complex lipid called phosphorylated dihydroceramides has been discovered to be of inflammatory activators. The bacterial lipids stimulate prostaglandin E2, IL-6, and TNF-α secretion, inhibit osteoblast differentiation and function, and induce osteoclast formation. The biological activities are in Toll-like receptor 2 (TLR2)-dependent manner. These new findings imply that bacterial lipids could be important virulent factors that cause apical bone resorption. Future investigations may determine the significance of the bacterial lipids in the pathogenesis and treatment of endodontic diseases.

Novel bacterial complex lipids called phosphorylated dihydroceramides have been recently discovered to be inflammatory activator. In this review the regulation of osteoblast and osteoclast differentiation and Toll-like receptors will be first introduced, as the activities of the bacterial lipids involve these two fields. Subsequently, recent and fresh findings of the biological activities of bacterial lipids and the implications for the pathogenesis of apical periodontitis will be summarized.

## Regulation of osteoblast and osteoclast differentiation and bone resorption

One of the characteristics of apical periodontitis is apical bone resorption. Bone is continuously remodeling to maintain bone volume and calcium homeostasis. Osteoblasts secrete and deposit bone matrix proteins including type I collagen, osteocalcin (OC), osteopontin (OPN), bone sialoprotein (BSP) and proteoglycans [1]. Osteoblasts also regulate the formation of hydroxyapatite crystals with high enzyme activity of alkaline phosphatase (ALP) [1]. Bone morphogenetic proteins (BMPs) are the key regulators of the differentiation of osteoblasts. BMPs enhance the expression of type I collagen, OC, and ALP [1]. Transcription factor Runx2 is the key regulator of osteoblast differentiation by binding Runx consensus sequence in the promoter of all major osteoblast genes [2][3]. BMP2 promotes Runx2 and Osterix expression [1]. Osterix, activating transcription factor 4 (ATF4), and β-Catenin, which act downstream of Runx2, also play essential roles in osteoblast differentiation [2]. In osterix deficient mice, there are no osteoblast differentiation and bone formation [4]. Late-stage osteoblast differentiation, which is represented by gene expression of OC and BSP, is significantly inhibited by inactivation of c-jun N-terminal kinase (JNK) and down-regulation of ATF4 [5]. Inactivation of β-Catenin stops mesenchymal progenitors differentiate to osteoblasts [6].

Osteoclasts are large multinucleated specialized giant cells responsible for bone resorption that arise from a hematopoietic stem cell lineage of monocytes and macrophages [7]. Progenitor cells undergo a series of differentiation stages during the development of osteoclastogenesis [8]. RANKL (receptor activator of nuclear factor kappa B ligand) and M-CSF (macrophage colony stimulating factor) are considered to be essential environmental factors during normal osteoclastogenesis [8][9]. Osteoclast differentiation is mediated by RANKL/RANK/OPG axis [10][11]. RANKL is expressed primarily on cell surface of bone marrow stromal cells and osteoblasts. RANK (receptor activator of nuclear factor kappa B) is mainly expressed in osteoclast precursors and mature osteoclasts. RANKL binds to its receptor (RANK) and activates several downstream signaling cascades leading to activation of several critical transcription factors that are required for osteoclast differentiation and activity [12]. Transcription factor NFATc1 is the crucial regulator of osteoclastogenesis and stimulates osteoclast specific genes, such as tartrate resistant acid phosphatase (TRAP) and cathepsin K (CSK) [13]. OPG (osteoprotegerin) is a protein that is secreted by osteoblasts. It binds to RANKL, occupies the binding site for RANK, and blocks its function. Mice deficient in RANK or RANKL have severe osteopetrosis with decreased osteoclasts [10][12][14]. Mice deficient in OPG have profound osteoporosis with increased osteoclastogenesis [15]. The expression of RANKL and OPG is highly modulated by multiple osteotropic agents and cytokines to tightly regulate osteoclast formation, activity, and survival [11][16]. In states of inflammation, RANKL expression can be significantly up-regulated by osteoblasts in response to pro-inflammatory cytokines [17].

M-CSF stimulates the expression of RANK on osteoclast precursors. M-CSF deficient mice are osteopetrotic due to defective osteoclast differentiation and activity [18][19]. Recent findings support the notion that LPS and some inflammatory cytokines such as TNF-α and IL-1 may also be directly involved in osteoclast differentiation and activation through a mechanism partially independent from that of RANKL-RANK interaction. TNF-α and IL-1 act through their own respective receptors [16][20][21].

## Toll-like Receptors (TLRs) and bacterial virulent factors

The innate immune system is the first line of host defense against pathogens. It recognizes microorganisms via pattern-recognition receptors (PRRs) [22]. TLRs are one of several classes of PRRs [23]. PRRs recognize microbial components, which are broadly shared by pathogens but distinguishable from host molecules, referred to as pathogen-associated molecular patterns (PAMPs) [22][23]. TLRs are expressed on various cells including macrophages, dendrite cells, B cells, certain types of T cells, fibroblasts, epithelial cells, and bone cells [22][23]. To date, 13 TLRs have been identified [24]. TLR4 is the LPS receptor. TLR2 recognizes a variety of microbial components, including lipoteichoic acid, lipoproteins, and peptidoglycan [22][23]. Interaction of TLRs with their specific PAMPs induces the secretion of pro-inflammatory cytokines and expression of co-stimulatory molecules [22][23][25].

## Biological activity of novel complex lipids and implications for the pathogenesis of apical periodontitis 

Pulp infections initially produce an inflammatory response within the pulp that often leads to complete pulpal necrosis and subsequently in the apical region, which results in apical lesion formation [26]. The significance of the role of microorganisms in the pathogenesis of pulpal and apical disease has been established by several classical studies [27][28][29][30]. The development of anaerobic techniques and modern molecular methods for culturing, detecting, and characterizing organisms have revealed that the bacteria involved in primary endodontic infections are predominantly Gram-negative anaerobic species [31][32][33][34][35].

The black-pigmented Gram-negative anaerobic bacteria such as Porphyromonas gingivalis (P.gingivalis), Porphyromonas endodontalis (P.endodontalis), Prevotella intermedia (P.intermedia) and Prevotella nigrescens (P.nigrescens) are relatively common in infected root canals and endodontic abscesses [32][36]. Their presence in root canals or abscesses has been confirmed by traditional culture and molecular genomic methods [37][38][39]. P.gingivalis and P.endodontalis have been closely associated with acute symptoms of endodontic infections [40][41]. P.gingivalis and Fusobacterium nucleatum (F.nucleatum) have been detected as co-colonizers in biofilms associated with apical lesions [42]. Recent studies have shown that P.gingivalis enhances biofilm formation by F.nucleatum, while F. nucleatum enhances attachment of P. gingivalis to the host cells [43][44]. Their synergistic relationship is important in polymicrobial endodontic infections.

Endodontopathogenic products of black-pigmented anaerobic bacteria may include fimbriae, cell capsule, outer membrane proteins, and endotoxic lipopolysaccharides [45]. LPS is one of the most studied microbial initiators of inflammation and endodontic pathogenesis [46][47][48][49][50][51][52][53]. LPS binds the TLR4/CD14/MD2 receptor complex and promotes the secretion of pro-inflammatory cytokines, such as TNF-α, IL-1, and IL-6 in many cell types, especially in macrophages [54][55]. Calcium hydroxide hydrolyzes the lipid moiety of LPS and alters the biological properties of LPS [56][57]. Recently, it has been discovered that black pigmented bacteria synthesizes complex lipids, comprising a group of unusual sphingolipids called dihydroceramides that possess impressive capacity to stimulate the secretion of inflammatory cytokines, inhibit osteoblast differentiation, and promote osteoclastogenesis [58][59][60].

Several novel complex lipids produced by P.gingivalis, termed phosphorylated dihydroceramides have been identified [61][62]. Three major classes of the ceramides are free (non-phosphorylated) dihydroceramides (DHC), phosphoglycerol dihydroceramide (PG DHC), and phosphoethanolamine dihydroceramide (PE DHC) [62]. These lipids are also recovered from calculus-contaminated root surfaces [63][64] and from diseased periodontal tissues [65]. These sphingolipids potentiate interleukin-1β (IL-1β) mediated secretion of inflammatory mediators from fibroblasts, including prostaglandin E2, and alter gingival fibroblast morphology and adherence [61][66]. Prostaglandin E2 is known to promote vasodilation, inflammatory responses and to stimulate osteoclast mediated bone resorption. Phosphoglycerol dihydroceramide is found to induce the apoptosis of human endothelial cells, which potentially account for the loss of attachment associated with periodontitis [67]. The phosphorylated dihydroceramides, particularly the PE DHC, enhance the experimental allergic encephalomyelitis in the murine model of multiple sclerosis, and induce dendritic cell interleukin-6 secretion in a TLR2-dependent manner [58]. These findings demonstrated that dihydroceramides may play important role in autoimmune diseases [58].

Furthermore, P.gingivalis lipids inhibit osteoblast differentiation in a concentration-dependent manner. However, P. gingivalis lipids do not significantly alter osteoblast proliferation, viability, or apoptosis [60]. Real time PCR shows down-regulation of osteoblast genes including Runx2, ALP, OC, BSP, OPG and DMP-1 with concurrent up-regulation of RUNKL, TNF-α, and MMP-3 genes [60]. The inhibitory effect of P.gingivalis lipids on osteoblast differentiation is attributed primarily to the PG DHC lipids and is shown to be dependent on TLR2 expression [60]. Moreover, P.gingivalis lipids inhibit calvarial osteoblast gene expression and they act in-vivo [60].

P.endodontalis produces analogous phosphorylated ceramide lipids as P.gingivalis with the exception of PG DHC, which P. endodontalis does not generate [68]. Like P.gingivalis lipids, P.endodontalis lipids inhibit osteoblast differentiation through engagement of TLR2 [68]. P.endodontalis and P.gingivalis lipids induce monocyte TNF-α secretion. Anti-TLR2, not anti-TLR4, antibody significantly reduced the effect of the lipids on monocytes as shown with the reduction of TNF-α production [68][69]. P.endodontalis and P.gingivalis lipids also promote osteoblast cell differentiation and maturation [68][69]. Again TLR2 antibody significantly reduced the number of the TRAP-positive multinucleated osteoclasts, which were induced by P.endodontalis and P.gingivalis lipids [69]. The presence of the bacteria lipids in the infected root canals was investigated with the control of vital pulp tissues. The phosphoethanolamine dihydroceramide associated with P.endodontalis was identified in the necrotic pulps [69].

## CONCLUSION

In summary, the bacterial lipids stimulate inflammatory mediator secretion, such as prostaglandin E2, IL-6, and TNF-α, inhibit osteoblast differentiation and function, and induce osteoclast formation. The lipids were also identified from infected root canals. These findings imply that the bacteria lipids could be virulent factors for the pathogenesis of apical periodontitis.

Future studies may determine the correlation between the bacteria lipids in root canals and the presence of apical lesions, the persistence of bacteria lipids in apical lesions, and the molecular mechanisms by which bacterial lipids cause apical bone resorption.
